# Self-perceived ability to cope with stress and depressive mood without smoking predicts successful smoking cessation 12 months later in a quitline setting: a secondary analysis of a randomized trial

**DOI:** 10.1186/s12889-018-5973-9

**Published:** 2018-08-28

**Authors:** Eva Nohlert, John Öhrvik, Ásgeir R. Helgason

**Affiliations:** 1 Centre for Clinical Research, Uppsala University, Hospital of Vastmanland Vasteras, 721 89 Vasteras, Sweden; 2 Department of Public Health Sciences, Social Medicine, Karolinska Institutet, Stockholm, Sweden; 3 Reykjavik University and Icelandic Cancer Society, Reykjavik, Iceland

**Keywords:** Depressive mood, Point prevalence, Self-efficacy, Self-perceived abilities, Single-item assessment, 6-month continuous abstinence, Tobacco

## Abstract

**Background:**

Telephone-based smoking cessation services (‘quitlines’) are both effective and cost-effective. Knowledge of modifiable baseline factors in real-life settings with heterogeneous participants is essential for the development and improvement of treatment protocols to assist in telephone-based smoking cessation. The aim was to assess if self-perceived abilities to cope measured at baseline, would predict abstinence at the 12-month follow-up at the Swedish National Tobacco Quitline (SNTQ).

**Methods:**

The data were retrieved from a previous randomized controlled trial comparing the effectiveness of proactive and reactive service at the SNTQ. Included were 612 clients calling the SNTQ between February 2009 and September 2010. Outcome measures were self-reported point prevalence and 6-month continuous abstinence at the 12-month follow-up. Plausible predictors of smoking cessation were assessed at the first call and in a baseline questionnaire. Self-perceived abilities at baseline were measured by two questions: (1) How likely is it that you will be smoke-free in one year? and (2) How likely are you to be able to handle stress and depressive mood without smoking? The associations between potential predictors and outcome (smoke-free at 12-month follow-up) were assessed by logistic regression analysis.

**Results:**

Of the two potential predictors for abstinence at 12-month follow-up, only the perceived ability to handle stress and depressive mood without smoking remained significant in the adjusted analyses (Odds Ratio, OR 1.13, 95% CI 1.00–1.27 for point prevalence and OR 1.16, 95% CI 1.01–1.33 for 6-month continuous abstinence according to intention-to-treat). The overall strongest predictor in the adjusted analyses was smoking status in the week before baseline (OR 3.30, 95% CI 1.79–6.09 for point prevalence and OR 3.97, 95% CI 2.01–7.83 for 6-month continuous abstinence).

**Conclusions:**

The perceived ability to handle stress and depressive mood without smoking at baseline predicted the subjects’ abstinence at the 12-month follow-up. An assessment of/adjustment for stress and depressive mood coping skills may be appropriate in future smoking cessation treatment and research. The treatment protocol can be tailored to individual differences and needs for optimal support.

**Trial registration:**

ClinicalTrials.gov: NCT02085616. Registered March 10, 2014, ‘retrospectively registered’.

## Background

Tobacco smoking is still one of the leading risk factors for early death and morbidity and is projected to kill more than eight million people per year by 2030 [1]. Non-communicable diseases (NCDs) are increasing worldwide and caused 40 million deaths in 2015 [2]. Tobacco smoking is a major cause of many NCDs and contributed to almost 150 million global disability-adjusted life-years (DALYs) in 2015 [3].

There is sound evidence for the efficacy of interventions and strategies to reduce tobacco consumption [4–7]. However, it is important to understand which factors are associated with quitting success for improving the efficacy of the interventions. Among smoking cessation therapies, telephone-based smoking cessation services (‘quitlines’) are both effective and cost-effective [7–12]. Knowledge of modifiable baseline factors in real-life settings with heterogeneous participants is essential for the development and improvement of treatment protocols in telephone-based smoking cessation.

Commonly reported predictors for successful quitting include high motivation, low nicotine dependence/low number of cigarettes smoked per day, high socio-economic status, social support, number and length of previous quit attempts, high self-efficacy to quit, low stress level, and no psychiatric comorbidity [7, 13, 14]. *Intention to quit* has been shown to predict quitting attempt [15–17]. In a review from 2011, the authors found motivational factors (such as intention to quit or wish to quit) to be associated with quitting attempts, but not consistently associated with maintaining abstinence [14]. *Stress and depressive mood* have also been associated with smoking behaviour and the ability to quit smoking in several studies [18–21]. In a previous prospective study using epidemiological methods at the Swedish National Tobacco Quitline (SNTQ), the authors found that clients who experienced periods of depressive mood and/or periods of stress after their first contact with SNTQ were less likely to be smoke-free at follow-up [13]. Thus, perceived ability to handle stress and depressive mood without smoking could be a predictor of quitting that may be modifiable during the treatment. In a RCT from 2014, the authors found that a stress and anger management programme significantly enhanced smoking cessation rates [22].

*Self-efficacy,* confidence in perceived ability to quit smoking and conceivable strategies to handle different situations without smoking, have been of interest for tobacco cessation research. High self-efficacy to quit smoking has been shown to predict successful quitting [23–27] but research on different aspects of self-efficacy and smoking cessation in a quitline setting is limited. In a randomized controlled trial (RCT) of quitline counselling versus the use of a self-help brochure for smoking parents, increased self-efficacy to refrain from smoking in stressful and tempting situations, and increased acceptance of craving to smoke significantly mediated the effect of smoking cessation counselling on prolonged abstinence at a 12-month follow-up with an explained variance of 25.1% [28]. One study found that low self-efficacy to quit smoking could predict relapse during 6 months after calling a quitline [29]. However, a meta-analysis from 2009 [30] found the association between self-efficacy and smoking abstinence to be greatly reduced when controlling for smoking status at the time of the assessment of the clients’ perceived ability to stop smoking.

The SNTQ was established in 1998 and is a free nationwide service operated by the Stockholm County Council Health Service and funded by the Swedish Government. In previous studies at the SNTQ, point prevalence abstinence rates have gradually increased from 28% to approximately 40%, using the definition of the study base as those responding to a baseline questionnaire [11, 13, 31]. The treatment protocol is a mixture of motivational interviewing, cognitive behaviour therapy, and pharmacological consultation. It has been a step-by-step process to identify modifiable baseline factors associated with quitting and to integrate them into the SNTQ support protocol [11, 13, 31].

In the present study we aimed to assess aspects of self-perceived abilities that may potentially be affected during treatment, and their relationship to successful cessation at 12-month follow-up at the SNTQ. We assessed i) self-perceived ability to quit smoking and ii) self-perceived ability to cope with stress and depressive mood without smoking. The rational for assessing these two aspects is mainly based on our clinical experience and our previous studies [13]. We do not claim to be using psychometrically validated methods to measure “self-efficacy”. However, the methodology used to assess self-perceived abilities in the present study is similar to that used to assess self-efficacy, which originates from Social Cognitive Theory [32]. Thus, we deem it appropriate to discuss our results in the context of self-efficacy research.

We hypothesized that high scores on clients’ own self-perceived ability to cope at baseline would predict abstinence at a 12-month follow-up. We tested this hypothesis for two different aspects of self-perceived ability to cope at baseline: (1) the likelihood of being smoke-free in 1 year and (2) the perceived ability to handle stress and depressive mood without smoking.

## Methods

### Standard SNTQ process

All calls to the SNTQ are registered in a computerized database. When a tobacco user calls to discuss his/her personal tobacco behaviour, the counsellor asks whether the client would like to sign up for cessation support. If the client gives verbal consent, their preference for call-back (proactive service) or no call-back (reactive service) is recorded, and a registration form including the baseline questionnaire, is mailed to them. The return of the baseline questionnaire is regarded as informed consent and the client is included in a study base to assess effectiveness. Twelve months after the first call, a follow-up questionnaire is sent by mail to the client. Non-responders to the follow-up questionnaire receive up to two reminders, one by mail, and one by telephone. The SNTQ and the counselling process have been described previously [11, 13, 31].

### Study population

**Fig. 1:**
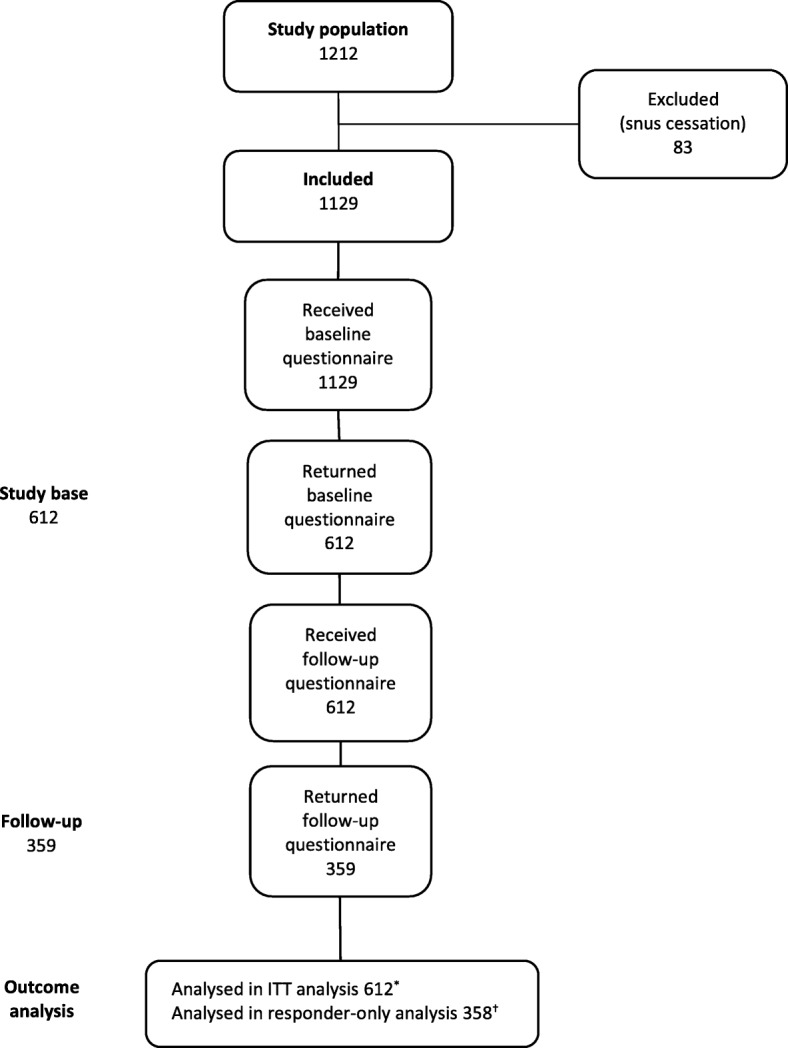
Flow chart of the study. * ITT, intention-to-treat † Internal drop-out for outcome variables in the follow-up questionnaire for one individual

The study was performed as part of the normal operation at the SNTQ, during a 20-month period in 2009–2010 when a RCT was carried out, with the primary aim to assess the effectiveness of the proactive versus the reactive service [11]. Thus, the clients were randomized to proactive or reactive services instead of being offered a choice. We included all clients recruited for tobacco cessation support at the quitline from February 2009 to September 2010. During that period, a total of 1212 calls were classified as new treatment calls. Snus (moist snuff) cessation calls were excluded, leaving 1129 in the study population. Those 612 subjects who returned the baseline questionnaire constituted the study base and were included in the Intention-To-Treat (ITT) analyses. The 359 who returned the follow-up questionnaire were included in responder-only analyses (Fig. 1).

### Questions and outcome measures

Baseline self-perceived ability to cope was assessed through two different questions: (1) ‘How likely is it that you will be completely smoke-free in one year?’ and (2) ‘I can handle stress and depressive mood without smoking’*.* The participants were asked to rate themselves on a numeric rating scale from 1= ‘not at all likely’ to 10 = ‘very likely’. To determine whether the response alternatives should be treated as numerical values or grouped/dichotomized, we analysed the proportion of quitters within each of the 10 steps in the scales and then plotted the results. We found nothing that supported a grouping/dichotomization, so the two self-perceived ability to cope questions were treated as numerical variables.

*Abstinence* at 12-month follow-up was assessed through two questions: (1) ‘Have you smoked (one or more deep drags) during the past 7 days?’ with response options of ‘no, not at all’, ‘yes, but not daily’, and ‘yes daily’; and (2) ‘When did you take your last puff?’ with response options of ‘0–7 days ago’, ‘more than 7 days but less than 6 months ago’, ‘6–12 months ago’, and ‘more than 12 months ago’. *Outcome measures* were point prevalence abstinence (not a puff in the previous 7 days) and 6-month continuous abstinence (not a puff in the previous 6 months) at the 12-month follow-up.

Additional questions comprised items about the daily consumption of cigarettes, snus, and tobacco cessation medication, different aspects of present and previous smoking habits and quitting attempts, willingness to use evidence based medication to overcome craving, exposure to second-hand smoke, and access to other support (social, professional).

The study was approved by the Ethics Committee at Karolinska Institutet (Dnr 00–367).

### Data analysis

IBM SPSS Statistics (version 24; IBM Corp., Armonk, NY, USA) was used to conduct the statistical analyses and *p* < 0.05 (two-sided) was considered statistically significant. Spearman’s rank correlation coefficient (rho) was calculated to assess any association between the two variables measuring self-perceived ability to cope. Logistic regression analysis was performed to calculate odds ratios (ORs) with 95% confidence intervals (CIs) for the two abstinence measures. Univariable analyses were performed for all relevant independent baseline variables. Established confounders described in the literature (gender, number of smoked cigarettes/day, smoking status at baseline, socio-economic status measured as education level, exposure to passive smoking, pharmaceutical use, snus use, and any other support) and the two self-perceived ability to cope variables were included in the multivariable analyses. The proportion of total variability explained by the model was assessed by Nagelkerke’s *R*^2^. The Hosmer and Lemeshow goodness-of-fit test was used to test the overall fit of the logistic regression model [33].

## Results

Baseline characteristics of the study population are listed in Table 1. Fifty-nine per cent (359/612) responded to the 12-month follow-up questionnaire. At the 12-month follow-up, the self-reported responders-only point prevalence abstinence was 46% (166/358) and the 6-month continuous abstinence rate was 35% (126/358) (Table 2).

**Table 1:** Population characteristics at baseline

	Total	Responders	Non-responders
Gender: women (% n/N)	77 (474/612)	77 (278/359)	77 (196/253)
Age groups, years (% n/N)			
≤ 34	20 (122/602)	16 (57/352)	26 (65/250)^a^
35–49	25 (150/602)	24 (85/352)	6 (65/250)
50–64	38 (231/602)	41 (146/352)	34 (85/250)
≥ 65	16 (99/602)	18 (64/352)	14 (35/250)
Number of years of education (md, q1-q3, N)	12, 10–13, 594	12, 9–14, 355	12, 10–13, 239
Number of smoked cigarettes/day (data journal) (md, q1-q3, N)	10, 0–20, 505	8, 0–15, 297	11, 3–20, 208^a^
No smoking in the week before baseline (% n/N)	28 (171/609)	34 (121/359)	20 (50/250)^a^
Number of years smoked before baseline (md, q1-q3, N)	32, 17–40, 575	35, 20–43, 344	30, 15–40, 231^a^
Snus use the week before baseline (% n/N)	7 (38/552)	6 (18/318)	9 (20/234)
Exposed to passive smoking (baseline) (% n/N)	26 (147/573)	22 (74/336)	31 (73/237) ^a^
Drug use (NRT, Zyban®, Champix®) the week before baseline (% n/N)	52 (308/592)	57 (198/349)	45 (110/243) ^a^
Other support at baseline (% n/N)	71 (428/600)	73 (254/349)	69 (174/251)
Likelihood of being smoke-free in 1 year (baseline, 1–10) (md, q1-q3, N)	8, 7–10, 590	9, 7–10, 347	8, 7–10, 243
Handle stress and depression successfully without smoking (baseline, 1–10) (md, q1-q3, N)	7, 4–9, 590	7, 4–9, 346	6, 4–8, 244 ^a^
Will use pharmaceuticals if necessary (baseline,1–10) (md, q1-q3, N)	10, 5–10, 592	10, 5–10, 348	9, 5–10, 244

^a^Statistically significant difference between responders and non-responders. Tested by Chi-2/Fisher’s Exact test or Mann-Whitney U-test

**Table 2:** Point prevalence and 6-month continuous abstinence at 12-month follow-up

% (n/N)	Point prevalence abstinence	6-month continuous abstinence	Corrected point prevalence abstinence ratio^b^	Corrected 6-month continuous abstinence ratio^b^
Responder-only	46.4 (166/358)	35.2 (126/358)	37.1	28.2
Intention to treat^a^	27.1 (166/612)	20.6 (126/612)	21.7	16.5

^a^ Non-responders at 12-month follow-up treated as smokers

^b^According to a proposed conservative correction factor of 0.8 for self-reported abstinence based on a non-response analysis at the SNTQ [47]

Results from the univariable logistic regression analyses are presented in Tables 3 and 4. The two baseline variables used to assess self-perceived ability to cope, the likelihood of being smoke-free in 1 year and perceived ability to handle stress and depressive mood without smoking were both significant predictors for abstinence.

**Table 3:** Univariable logistic regression analyses for responders-only point prevalence and 6-month continuous abstinence

Point prevalence abstinence			
Variable	n/N^a^	OR (95% CI for OR)	*p*
Gender; men vs. women (ref)	81/358 vs. 277/358	0.53 (0.32–0.89)	0.016
Age, years			0.077
- ≤ 34 (ref)	57/351	1.0	
- 35-49	85/351	1.22 (0.62–2.39)	0.559
- 50-64	145/351	0.94 (0.51–1.74)	0.844
- ≥ 65	64/351	0.51 (0.24–1.06)	0.070
Number of smoked cigarettes/day (data journal)	md = 8, q_1_ = 0, q_3_ = 15, *N* = 296	0.94 (0.92–0.97)	< 0.001
Variables from baseline questionnaire			
Number of years of education	md = 12, q_1_ = 9, q_3_ = 14, *N* = 354	1.07 (0.99–1.16)	0.067
Number of years smoked before baseline	md = 35, q_1_ = 20, q_3_ = 43, *N* = 343	0.99 (0.97–1.00)	0.076
Smoking the week before baseline; no vs. yes (ref)	121/358 vs. 237/358	4.29 (2.68–6.87)	< 0.001
Passive smoking at baseline; not exposed vs. exposed (ref)	261/335 vs. 74/335	1.37 (0.81–2.31)	0.240
Drug use (NRT, Zyban®, Champix®) the week before baseline; yes vs. no (ref)	197/348 vs. 151/348	1.48 (0.97–2.27)	0.073
Snus use the week before baseline; yes vs. no (ref)	18/317 vs. 299/317	0.41 (0.14–1.19)	0.102
Other support at baseline: yes^b^ vs. no (ref)	254/348 vs. 94/348	1.79 (1.10–2.92)	0.019
Likelihood of being smoke-free in 1 year (baseline, 1–10)	md = 9, q_1_ = 7, q_3_ = 10, *N* = 346	1.26 (1.13–1.41)	< 0.001
Handle stress and depression successfully without smoking (baseline, 1–10)	md = 7, q_1_ = 4, q_3_ = 9, *N* = 345	1.22 (1.13–1.33)	< 0.001
Will use pharmaceuticals if necessary (baseline,1–10)	md = 10, q_1_ = 5, q_3_ = 10, *N* = 347	0.98 (0.91–1.04)	0.476
6-month continuous abstinence			
Variable	n/N^a^	OR (95% CI for OR)	*p*
Gender; men vs. women (ref)	81/358 vs. 277/358	0.62 (0.36–1.07)	0.087
Age, years			0.070
- ≤ 34 (ref)	57/351	1.0	
- 35-49	85/351	1.50 (0.75–2.99)	0.254
- 50-64	145/351	1.00 (0.53–1.91)	0.991
- ≥ 65	64/351	0.57 (0.26–1.25)	0.160
Number of smoked cigarettes/day (data journal)	md = 8, q_1_ = 0, q_3_ = 15, *N* = 296	0.94 (0.91–0.97)	< 0.001
Variables from baseline questionnaire			
Number of years of education	md = 12, q_1_ = 9, q_3_ = 14, *N* = 354	1.06 (0.98–1.15)	0.145
Number of years smoked before baseline	md = 35, q_1_ = 20, q_3_ = 43, *N* = 343	1.00 (0.98–1.01)	0.572
Smoking the week before baseline; no vs. yes (ref)	121/358 vs. 237/358	5.28 (3.28–8.49)	< 0.001
Passive smoking at baseline; not exposed vs. exposed (ref)	261/335 vs. 74/335	1.87 (1.04–3.37)	0.037
Drug use (NRT, Zyban®, Champix®) the week before baseline; yes vs. no (ref)	197/348 vs. 151/348	1.28 (0.82–2.00)	0.278
Snus use the week before baseline; yes vs. no (ref)	18/317 vs. 299/317	0.21 (0.05–0.93)	0.039
Other support at baseline: yes^b^ vs. no (ref)	254/348 vs. 94/348	1.53 (0.92–2.56)	0.103
Likelihood of being smoke-free in 1 year (baseline, 1–10)	md = 9, q_1_ = 7, q_3_ = 10, *N* = 346	1.39 (1.21–1.59)	< 0.001
Handle stress and depression successfully without smoking (baseline, 1–10)	md = 7, q_1_ = 4, q_3_ = 9, *N* = 345	1.29 (1.18–1.41)	< 0.001
Will use pharmaceuticals if necessary (baseline,1–10)	md = 10, q_1_ = 5, q_3_ = 10, *N* = 347	0.96 (0.90–1.03)	0.264

^a^ n = number in category, N = total number in analysis

^b^ Social and/or professional support

**Table 4:** Univariable logistic regression analyses for point prevalence and 6-month continuous abstinence, including non-responders at follow-up treated as smokers

Point prevalence abstinence			
Variable	n/N^a^	OR (95% CI for OR)	*p*
Gender; men vs. women (ref)	138/612 vs. 474/612	0.62 (0.39–0.98)	0.041
Age, years			0.203
- ≤ 34 (ref)	122/602	1.0	
- 35-49	150/602	1.49 (0.86–2.56)	0.156
- 50-64	231/602	1.43 (0.86–2.37)	0.167
- ≥ 65	99/602	0.90 (0.48–1.71)	0.757
Number of smoked cigarettes/day (data journal)	md = 10, q_1_ = 0, q_3_ = 20, *N* = 505	0.94 (0.91–0.96)	< 0.001
Variables from baseline questionnaire			
Number of years of education	md = 12, q_1_ = 10, q_3_ = 13, *N* = 594	1.05 (0.99–1.12)	0.116
Number of years smoked before baseline	md = 32, q_1_ = 17, q_3_ = 40, *N* = 575	1.00 (0.99–1.01)	0.957
Smoking the week before baseline; no vs. yes (ref)	171/609 vs. 438/609	4.19 (2.85–6.15)	< 0.001
Passive smoking at baseline; not exposed vs. exposed (ref)	426/573 vs. 147/573	1.64 (1.04–2.57)	0.032
Drug use (NRT, Zyban®, Champix®) the week before baseline; yes vs. no (ref)	308/592 vs. 284/592	1.72 (1.19–2.49)	0.004
Snus use the week before baseline; yes vs. no (ref)	38/552 vs. 514/552	0.39 (0.15–1.02)	0.054
Other support at baseline: yes^b^ vs. no (ref)	428/600 vs. 172/600	1.73 (1.13–2.66)	0.012
Likelihood of being smoke-free in 1 year (baseline, 1–10)	md = 8, q_1_ = 7, q_3_ = 10, *N* = 590	1.23 (1.11–1.36)	< 0.001
Handle stress and depression successfully without smoking (baseline, 1–10)	md = 7, q_1_ = 4, q_3_ = 9, *N* = 590	1.22 (1.13–1.31)	< 0.001
Will use pharmaceuticals if necessary (baseline,1–10)	md = 9, q_1_ = 5, q_3_ = 10, *N* = 592	0.99 (0.93–1.04)	0.624
6-month continuous abstinence			
Variable	n/N^a^	OR (95% CI for OR)	*p*
Gender; men vs. women (ref)	138/612 vs. 474/612	0.67 (0.41–1.12)	0.127
Age, years			0.144
- ≤ 34 (ref)	122/602	1.0	
- 35-49	150/602	1.73 (0.95–3.17)	0.075
- 50-64	231/602	1.45 (0.82–2.56)	0.207
- ≥ 65	99/602	0.91 (0.44–1.89)	0.801
Number of smoked cigarettes/day (data journal)	md = 10, q_1_ = 0, q_3_ = 20, *N* = 505	0.93 (0.91–0.96)	< 0.001
Variables from baseline questionnaire			
Number of years of education	md = 12, q_1_ = 10, q_3_ = 13, *N* = 594	1.05 (0.98–1.12)	0.188
Number of years smoked before baseline	md = 32, q_1_ = 17, q_3_ = 40, *N* = 575	1.01 (0.99–1.02)	0.423
Smoking the week before baseline; no vs. yes (ref)	171/609 vs. 438/609	5.41 (3.56–8.21)	< 0.001
Passive smoking at baseline; not exposed vs. exposed (ref)	426/573 vs. 147/573	2.14 (1.25–3.68)	0.006
Drug use (NRT, Zyban®, Champix®) the week before baseline; yes vs. no (ref)	308/592 vs. 284/592	1.54 (1.03–2.31)	0.035
Snus use the week before baseline; yes vs. no (ref)	38/552 vs. 514/552	0.20 (0.05–0.84)	0.028
Other support at baseline: yes^b^ vs. no (ref)	428/600 vs. 172/600	1.57 (0.99–2.52)	0.058
Likelihood of being smoke-free in 1 year (baseline, 1–10)	md = 8, q_1_ = 7, q_3_ = 10, *N* = 590	1.35 (1.19–1.53)	< 0.001
Handle stress and depression successfully without smoking (baseline, 1–10)	md = 7, q_1_ = 4, q_3_ = 9, *N* = 590	1.29 (1.19–1.41)	< 0.001
Will use pharmaceuticals if necessary (baseline,1–10)	md = 9, q_1_ = 5, q_3_ = 10, *N* = 592	0.97 (0.91–1.03)	0.365

^a^ n = number in category, N = total number in analysis

^b^ Social and/or professional support

These two self-perceived ability to cope variables were relatively strongly correlated (Spearman’s rho 0.522, *p* < 0.001). However, only the perceived ability to handle stress and depressive mood without smoking remained significant for abstinence at month 12 in the adjusted analyses, with an OR of 1.13 for point prevalence and 1.16 for 6-month continuous abstinence according to ITT (Table 5). The overall strongest predictor for abstinence in the adjusted analyses was smoking status in the week before baseline, with an OR of 3.30 for point prevalence and 3.97 for 6-month continuous abstinence (Table 5).

**Table 5:** Multivariable logistic regression analyses^a^ for point prevalence abstinence and 6-month continuous abstinence

*N* = 214 Responders-only				
Variable	Point prevalence abstinence^b^		6-month continuous abstinence^c^	
	OR (95% CI)	*p*	OR (95% CI)	*p*
Likelihood of being smoke-free in 1 year	1.00 (0.84–1.19)	0.985	1.04 (0.85–1.27)	0.695
Perceived ability to handle stress and depressive mood without smoking	1.18 (1.03–1.36)	0.017	1.20 (1.03–1.39)	0.020
Smoked in the week before baseline (ref. = yes)	3.28 (1.58–6.81)	0.001	3.93 (1.86–8.31)	< 0.001
*N* = 362 Including non-responders at follow-up (treated as smokers)				
Variable	Point prevalence abstinence^d^		6-month continuous abstinence^e^	
	OR (95% CI)	*p*	OR (95% CI)	*p*
Likelihood of being smoke-free in 1 year	1.02 (0.88–1.19)	0.762	1.05 (0.88–1.25)	0.570
Perceived ability to handle stress and depressive mood without smoking	1.13 (1.00–1.27)	0.050	1.16 (1.01–1.33)	0.033
Smoked in the week before baseline (ref. = yes)	3.30 (1.79–6.09)	< 0.001	3.97 (2.01–7.83)	< 0.001

Variables shown in the table are the two self-perceived ability to cope variables and statistically significant variables

^a^Included variables: gender, age, number of cigarettes/day, number of years of education, number of years smoked, smoking in the week before baseline, passive smoking, drug use, snus use, other support, likelihood of being smoke-free in 1 year, handle stress and depressive mood without smoking, and willingness to use pharmaceuticals

^b^Nagelkerke R^2^ 26.1%. Hosmer and Lemeshow test of goodness of fit, *p* = 0.356

^c^Nagelkerke R^2^ 28.2%. Hosmer and Lemeshow test of goodness of fit, *p* = 0.202

^d^Nagelkerke R^2^ 21.5%. Hosmer and Lemeshow test of goodness of fit, *p* = 0.193

^e^Nagelkerke R^2^ 24.5%. Hosmer and Lemeshow test of goodness of fit, *p* = 0.914

## Discussion

The participants’ perceived ability to handle stress and depressive mood without smoking at baseline predicted abstinence at the 12-month follow-up when adjusted for all potential confounders. However, the likelihood of being smoke-free in 1 year did not.

As in previous studies, the strongest predictor for abstinence at the 12-month follow-up was smoking status in the week before baseline, a variable that can serve as a proxy for motivation as well as dependence [7, 13, 14]. A systematic review reported that motivational factors dominate the prediction of quit attempts, but that only cigarette dependence consistently predicts the success of those attempts in adult general populations [14] . Similar findings are reported from the International Tobacco Control Four Country Survey [15] and from the ATTEMPT cohort study [17]. One study reported that dependence, but not motivation, predicted abstinence in a clinical sample of smokers seeking help to quit [34].

Self-efficacy research, using similar scales as in the present study, has been associated with smoking abstinence in numerous studies [23–26, 28–30, 35–39]. Two multi-item self-efficacy measures¸ the “Smoking Abstinence Self-Efficacy Scale” [40] and the “Smoking Self-Efficacy Questionnaire” [41], have been frequently used in smoking cessation studies [25, 28, 36, 38]. Generally, multi-item measures are likely to be more reliable and valid than single-item measures in assessing different constructs. However, in one meta-analysis, the authors did not find support for their hypothesis that the association between self-efficacy and smoking cessation would be moderated by the number of items measuring self-efficacy [30].

We assessed two aspects of self-perceived ability to cope using single-item questions with response alternatives from 1 to 10 on a numeric rating scale. The items were aimed at assessing a specific situation during a specific period [32]. The perceived likelihood of being smoke-free in 1 year and perceived ability to handle stress and depressive mood without smoking were both statistically significant predictors of successful smoking cessation in the univariable analyses. However, in the multivariable analyses, only the participants’ perceived ability to handle stress and depressive mood without smoking remained significant (when adjusted for all potential confounders).

A single-item measure, ‘How confident are you that you will be able to quit for good at this time?’ on a 5-point scale was a reliable predictor of relapse in an Australian quitline study [29]. Lindberg et al. who used a single-item question, ‘How confident are you that you will succeed if you decide to quit?’, and a 10-point scale in a study of smoking patients with chronic obstructive pulmonary disease (COPD), found it to be a valuable instrument for predicting smoking cessation over several years [37]. Hendricks et al. also used a single-item question to measure self-efficacy as a predictor for abstinence in a RCT of a cognitive behavioural smoking cessation intervention among treatment-seeking smokers [24]. They asked the smokers to indicate on a 10-point scale how successful they expected to be in quitting.

Because of the clinical setting in our study, we deemed it more feasible to use single-item questions. We further discuss the single-item assessment below, under the heading ‘Strengths and limitations’.

Since the questions were only asked on one occasion, we could not assess whether changes in self-perceived abilities over time affected the progression of abstinence rates. However, in the multivariable analyses we controlled for the baseline smoking status. This is an important issue, because those who have already managed to quit on their own before calling the quitline could be expected to have a higher belief in their own ability to quit [30]. Intention and perceived ability to quit are dynamic factors and consequently predicting smoking cessation based on these factors can be difficult. Self-efficacy and intention to quit vary over time, even daily [23, 42, 43] and tend to differ between studies [16, 42, 44].

The timing of self-perceived ability assessment can be important. The relationship between self-efficacy and future smoking has been reported to be weaker when self-efficacy is assessed prior to a quit attempt and stronger when assessed after the subjects have quit smoking [30]. The authors of that meta-analysis concluded that controlling for smoking status at the time of the self-efficacy assessment substantially reduced the relationship. In a study of smoking parents in the Netherlands, increased self-efficacy to refrain from smoking in stressful and tempting situations significantly mediated the effect of quitline cessation counselling on prolonged abstinence at a 12-month follow-up. In that study, self-efficacy was assessed at 3 months post-measurement. The authors concluded that the effect could be a *result* of quitting rather than a mechanism that contributed to it [28].

### Strengths and limitations

Using single items has been criticized for a lack of sensitivity [32], and we acknowledge this methodological problem. However, both items actually predicted abstinence in the univariable analyses and one item remained statistically significant in the multivariable analyses.

In the present study, the self-perceived ability questions were asked on one occasion, in the baseline questionnaire to which the clients responded within 7 days after their first call to the quitline. Consequently, we cannot comment on possible changes in self-perceived ability during treatment and its possible relation to quit rates, which is a limitation. However, we asked for smoking status at the same time as self-perceived ability was assessed, which probably is a strength of the study.

The double-barrel nature of the stress/depressive mood question warrants caution. Although the question did predict smoking cessation outcome in its present form, we do not know whether assessing belief in ability to handle stress versus depressive mood separately might affect the outcome differently. Therefore, additional research is necessary to discover the potential independent predictive value of self-efficacy/self-perceived ability for ‘handling stress’ versus ‘handling depressive mood’ using single-item assessment.

Nicotine dependence is a sound predictor for successful quitting [14]. In the present study, we only obtained information on one of the two most important measures of dependence, namely, the number of smoked cigarettes per day at baseline, not the time to the first cigarette in the morning, which is a potential limitation.

The study was performed within the normal running activity at the SNTQ, and thus under real-world conditions, which might be a strength for generalizability. However, the results may be limited to Swedish-speaking smokers seeking treatment. Obviously, the response rate of only 59% at the 12-month follow-up is a limitation to the analysis. Although relatively normal for studies like this [45] it might be a potential source of bias. We attempted to control for this by comparing the proportion of smokers at baseline among the responders versus non-responders. The proportion of smokers was significantly higher among non-responders (80%) than responders (66%) (*p <* 0.001). Non-responders were also younger, more exposed to passive smoking, were less likely to use tobacco cessation medication (Nicotine Replacement Therapy (NRT), bupropion (e.g. Zyban®), varenicline (Champix®)), and had lower scores on ability to handle stress and depressive mood without smoking, than were responders (Table 1). A conservative way to handle a relatively low response rate is to report the non-responders as still smokers in a separate analysis together with the responder-only analyses. We thus publish both analyses.

Self-reported smoking cessation assessment may also potentially affect the validity of the outcome assessment in all smoking cessation studies. Yet, biochemical verification is not required and may not be desirable in studies where the optimal data collection methods are through mail, telephone, or the Internet [46]. Also, it is unlikely that the proportion of false positives would be different for different self-efficacy levels at baseline.

### Potential application of the present findings to the quitline protocol

At present the questions of self-perceived ability to cope are included in the baseline questionnaire. As it is theoretically possible to influence self- perceived ability to cope, it could be a benefit to assess the clients’ self-perceived ability to cope/self-efficacy already at the first call and adapt the support accordingly.

## Conclusions

Perceived ability to handle stress and depressive mood without smoking at baseline predicted abstinence at the 12-month follow-up. According to the results, an assessment of/adjustment for stress and depressive mood coping skills may be appropriate in future smoking cessation treatment and research. The treatment protocol can be tailored to individual differences and needs for optimal support.

## Abbreviations

CI: Confidence Interval
ITT: Intention-To-Treat
OR: Odds Ratio
SNTQ: Swedish National Tobacco Quitline
